# Leptin and Adiponectin: new players in the field of tumor cell and leukocyte migration

**DOI:** 10.1186/1478-811X-7-27

**Published:** 2009-12-23

**Authors:** Kerstin Lang, Janina Ratke

**Affiliations:** 1 Institute of Immunology, Witten/Herdecke University, Stockumer Str. 10, 58448 Witten, Germany

## Abstract

Adipose tissue is no longer considered to be solely an energy storage, but exerts important endocrine functions, which are primarily mediated by a network of various soluble factors derived from fat cells, called adipocytokines. In addition to their responsibility to influence energy homeostasis, new studies have identified important pathways linking metabolism with the immune system, and demonstrating a modulatory role of adipocytokines in immune function. Additionally, epidemiological studies underline that obesity represents a significant risk factor for the development of cancer, although the exact mechanism of this relationship remains to be determined. Whereas a possible influence of adipocytokines on the proliferation of tumor cells is already known, new evidence has come to light elucidating a modulatory role of this signaling substances in the regulation of migration of leukocytes and tumor cells. The migration of leukocytes is a key feature to fight cancer cells, whereas the locomotion of tumor cells is a prerequisite for tumor formation and metastasis. We herein review the latest tumor biological findings on the role of the most prominent adipocytokines leptin and adiponectin, which are secreted by fat cells, and which are involved in leukocyte migration, tumor growth, invasion and metastasis. This review thus accentuates the complex, interactive involvement of adipocytokines in the regulation of migration of both leukocytes and tumor cells, and gives an insight in the underlying molecular mechanisms.

## Introduction

The prevalence and incidence of obesity and obesity-related diseases has increased dramatically over the past decades. Globally, the overweight population has exceeded one billion, and epidemiologic data collected clearly demonstrate that obesity in adults is associated with an increased risk of cardiovascular disease, diabetes, and numerous other health disorders [1]. Traditionally, fat tissue was considered to be solely an energy storage depot having only a passive function in the body. However, recent studies have shown that fat tissue exerts important endocrine functions, which are orchestrated by a complex network of various soluble factors derived from adipocytes (fat cells), called adipocytokines. These are a group of novel and highly active molecules, which are abundantly secreted by adipocytes, and act at both the local and systemic level [2]. Since their discovery in the early 90s, around 20 members of the adipocytokine family have been identified so far [3]. Adiponectin and leptin are the most abundant adipocytokines produced by adipocytes, and the best-studied molecules in this class so far. In addition to their responsibility to influence energy homeostasis, new studies have identified important pathways linking metabolism with the immune system and vice versa. Obesity is associated with a state of chronic low level inflammation, which is characterized by abnormal cytokine production and the activation of pro-inflammatory signaling pathways. Above all, new evidence elucidates their modulatory role in regulating cancer development [3,4]. Epidemiological studies further support an increased risk of cancer in obese individuals [5-7], although the exact mechanisms and thus prospects for therapeutic intervention are still unidentified.

Cell migration is an essential characteristic of both physiological and pathological processes within the human body. The migratory capacity of immune cells is a mandatory component for host immune surveillance, in which e.g. leukocytes from the circulation migrate into the surrounding tissues to destroy invading microorganisms and infected cells, whereas the migration of tumor cells is a prerequisite for tumor cell invasion and metastasis formation. Migratory activity is not an intrinsic function of the cells, but a process that is regulated by extracellular signal substances from other tissues and organ systems within the body. We have repeatedly shown that external signal substances such as neurotransmitters and cytokines significantly stimulate the migration of tumor cells and leukocytes [8,9]. Although several effects of adipocytokines on cells of the immune system and tumor cells have been described before, it is not known how the migratory activity of these cells is affected and the participating molecules and pathways are not yet identified. So, being aware of the alarmingly number of obese patients worldwide, this review aims to illuminate the latest findings on the role of adipocytokines in leukocyte migration, tumor growth, invasion and metastasis.

Understanding this rapidly growing family of adipocytokines and their mode of action would represent a break-through research in obesity and cancer, and will open new avenues for future development of obesity- and cancer-associated therapeutics.

### Leptin - Effects on immune cells and tumor cells

A key molecule in obesity is leptin, a 16 kDa peptide hormone predominantly produced by white adipose tissue [10]. Circulating leptin is actively transported through the blood-brain barrier and acts on the hypothalamic satiety center to decrease food intake. The main function of leptin in the human body is the regulation of energy expenditure and control of appetite. Indeed, lack of leptin in mice with a mutation in the gene encoding leptin, or absence of functional leptin receptor (*db/db *mice) results in obesity and many associated metabolic complications such as insulin resistance [11]. Serum level of leptin reflects the amount of energy stored in the adipose tissue and is in proportion to body fat mass [12], i.e. increased in obese and decreased after several months of pronounced weight loss [13].

Leptin acts via transmembrane receptors (OB-R), which belong to the class I cytokine receptor family, such as the receptors of interleukin-2 (IL-2), IL-3, IL-4, IL-6, IL-11, IL-12, granulocyte colony-stimulating factor (G-CSF) or leukemia inhibitory factor (LIF). The OB-R has at least six isoforms, termed OB-R(a-f), which are generated primarily by alternative splicing of the *ob *gene. They all share an identical extracellular ligand-binding domain but differ in their C-terminal regions [14]. Two main leptin receptor isoforms dominate: the short leptin receptor isoform (OB-Ra) and the long leptin receptor isoform (OB-Rb). OB-Rb contains the full-length intracellular domain and is believed to be the main leptin signaling receptor. OB-Rb has full signaling capabilities and is able to activate the JAK/STAT pathway, the major pathway used by leptin to exert its effects [15]. High levels of this isoform exist in the hypothalamus, but is represented on many other cell types as well, such as adipocytes, osteoclasts, endothelial cells, lung and kidney cells, mononuclear blood cells, muscle, endometrial and liver cells [14]. OB-Ra and OB-Rc are highly expressed in choroids plexus and microvessels, where they may play a role in leptin uptake or efflux from the cerebrospinal fluid as well as in receptor mediated transport of leptin across the blood-brain barrier [16]; OB-Re, which lacks the intracellular domain, may encode a soluble receptor. Consistent with leptin's role in controlling appetite and energy metabolism, OB-Rs have been found in the hypothalamus and adjacent brain regions. However, the almost universal distribution of OB-Ra and OB-Rb reflects the multiplicity of biological effects in extraneural tissues, providing evidence for the extreme functional pleiotropy of leptin [12].

Leptin plays important roles in both adaptive and innate immunity, and humans lacking leptin function exhibit impaired immunity. Accordingly, the leptin receptor is found to be expressed on a variety of immune cells [17]. With regard to innate immunity, leptin is a direct potent chemoattractant for monocytes and macrophages, whereas the presence of full-length receptors on migrating cells is required. In addition, leptin increases the recruitment of blood monocytes via adipose-tissue derived endothelial cells (EC) by stimulating the upregulation of EC adhesion molecules necessary for the diapedesis of the monocytes [18]. Acting on monocytes leptin induces the release of other cytokines such as tumor necrosis factor alpha (TNF-α) or interleukin-6 (IL-6) as well as CC-chemokine ligand 2 (CCL2) and vascular endothelial growth factor (VEGF) [19]. Leptin is also able to stimulate the chemokinesis of eosinophils [20], and the chemotaxis of neutrophils [21,22]. However, whereas leptin alone induces the migration of neutrophils and exerts by itself a chemoattractive effect comparable to that of well-known formyl-methionyl-leucyl-phenylalanin [21], neutrophil locomotion in response to classical chemoattractants is inhibited by simultaneous treatment with leptin [22]. Most of these effects are mediated through the OB-Rb, which is expressed mainly by endothelial cells and various leukocytes. In adaptive immunity, leptin enhances T cell proliferation and Th 1 proinflammatory cytokine production *in vitro*, whereas nothing is known about the effect of this adipocytokine on the migratory behaviour of T cells. Acting on dendritic cells leptin activates them, licenses them for Th 1 priming, and increases migratory performance [23].

Epidemiological studies have shown that obesity is a risk factor for postmenopausal breast cancer, cancers of the endometrium, colon and kidney, and malignant adenomas of the oesophagus [3]. Obese individuals have approximately a 1.5-3.5-fold increased risk of developing these cancers compared with normal-weight individuals, and it is estimated that 15 to 45% of these cancers are attributable to overweight (BMI 25.0-29.9 kg/m2) and obesity in Europe [24]. Moreover, in high income countries the attributable fraction of all cancers due to obesity was estimated as 3% [25].

Functional leptin receptors are found to be expressed on diverse cancer cells derived from different tissues such as breast, colon or prostate [26-30]. The breast cancer cell lines HTB-26 and ZR75-1 [27], the prostate cancer cell lines DU145 and PC-3 [30], and various colon carcinoma cell lines such as LS174T and HM7 [29] as well as SW480, SW620 and HCT116 [26] all express OB-Ra and OB-Rb. In breast cancer cell lines and in human primary breast carcinoma leptin receptor has been demonstrated to occur in combination with leptin. Therein, leptin is able to induce the growth of these cells via different pathways, can mediate angiogenesis by inducing the expression of VEGF, and promotes invasion and migration by transactivation of the epidermal growth factor receptor (EGFR) [15]. A bidirectional crosstalk between leptin and insulin-like growth factor-I (IGF-I) signaling was also shown to stimulate invasion and migration of breast cancer cells [31]. Thereby, IGF-I induced phosphorylation of Ob-Rb and leptin induced phosphorylation of IGF-I receptor, whereas cotreatment induced synergistic phosphorylation and association of Ob-Rb and IGF-IR along with activation of downstream effectors, Akt and extracellular signal-regulated kinase; in parallel this cotreatment synergistically transactivated EGFR. In 92% of breast carcinomas examined leptin was found to be overexpressed, but in none of the cases of normal breast epithelium. Remarkably, distant metastasis was present in 34% of OB-R-positive tumors with leptin overexpression, but in none of the cases where the tumors lacked OB-R expression or leptin overexpression [32]. A multitude of other studies have demonstrated that leptin mediates a significant increase of proliferation in breast, colon, esophagal and prostate cancer cells, too [3,27,30]. For example, Somasundar et al. [30] showed that leptin induced *in vitro *proliferation and inhibited apoptosis in DU145 and PC-3 prostate cancer cell lines. In a murine model of preneoplastic Apc(Min/+) colon epithelial cells leptin treatment was demonstrated to promote cell proliferation by an autocrine IL-6 production and trans-IL-6 signaling [33].

Moreover, leptin promotes the migration and invasion of cells derived from glioma [34], chondrosarcoma [35], colon carcinoma [29,36], hepatocellular and endometrial carcinoma cells [37,38], as well as prostate cancer [28]. In DU145 and PC-3 prostate cancer leptin significantly enhanced cell migration and induced expression of VEGF, transforming growth factor-beta1 (TGF-beta1), and basic fibroblast growth factor (bFGF), and thus overall likely contributes to the progression of prostate cancer [28]. Interestingly, Deo et al [39] observed differential effects of leptin on the invasive potential of prostate carcinoma cells depending on their androgen sensitivity. Androgen-sensitive LNCaP cells showed a significant increase in cellular proliferation upon treatment with leptin, whereas no effect was observable on androgen-insensitive PC-3 and DU145 cells. In contrast, leptin caused a significant dose-dependent decrease in migration and invasion solely of PC-3 and DU145 prostate carcinoma cell lines. These results are further supported by our own experiments demonstrating divergent effects of leptin on the proliferation and migration of carcinoma cells derived from different tissues (Table 1). Whereas leptin enhanced the proliferation of various breast carcinoma cell lines, including MDA-MB-468 and MDA-MB-231, it did not have any impact on the migratory activity of these cells. However, in various human colon carcinoma cells leptin significantly stimulated the locomotory behaviour of the cells [26,29]. These contradictory leptin effects on the migration and proliferation, especially of prostate and breast carcinoma cells, might be ascribable to the hormone-sensitivity of the cells. This conclusion is confirmed by results which demonstrate an influence of leptin on breast cancer development in relation to estrogen receptor status [40], and illuminate the growth-inducing effect of leptin in estrogen receptor-positive breast cancer cells by its stimulation of aromatase expression and the accompanied increase of estrogen levels through the aromatization of androgens [15]. In summary, all these data clearly support a direct functional role of leptin in processes related to cancer initiation and/or progression by promoting the migration and proliferation of carcinoma cells, thus resulting in metastatic development.

**Table 1:** Summary of the effects of leptin and adiponectin on migration and proliferation of various cancer cell lines

	Breast Carcinoma Cells			Colon Carcinoma Cells
	
	MCF-7	MDA-MB-468	MDA-MB-231	SW480
**Receptor expression**				

**ObRI**	+	+	+	+

**AdipoR1**	+	+	+	+

**AdipoR2**	+	+	+	+

**Migration**				

**Leptin**	no	no	no	+

**Adiponectin**	no	no	no	+

**Leptin+Adipo**.	no	no	no	-

**Proliferation**				

**Leptin**	+19%	+10%	+44%	+52%

**Adiponectin**	+25%	+18%	+46%	-31%

**Leptin+Adipo**.	+16%	+5%	+61%	+11%

Receptor expression was determined using flow cytometry, migratory activity was investigated using our three-dimensional cell migration assay, and cell proliferation after 96 hours was evaluated using well-established XTT assay. All methods were performed as described previously [9].

### Leptin - Molecular mechanisms

Leptin acts through its receptor OB-R, which is a member of the cytokine receptor family. Accordingly, leptin signaling is thought to be transmitted predominantly by the intracellular Janus kinase (JAK)-signal transducer and activator of transcription (STAT) pathway with the activation of STAT-3 and extracellular signal-regulated kinase (ERK)1/2 [3]. The engagement of OB-R by leptin leads to JAK-2 phosphorylation, which can then recruit STAT-3 tyrosine phosphorylation, finally leading to a nuclear translocation and stimulation of transcription. In addition, the leptin receptor is also known to intracellularly activate MAPK pathway after leptin binding [41]. In human endometrial and hepatocellular carcinoma cells leptin was shown to promote proliferation and invasion by rapidly stimulating the JAK/STAT-pathway and inducing the phosphorylation of ERK and AKT, thus activating these key signal transduction pathways associated with cell growth and cell migration [37,38,42]. Recent studies have shown that the ERK pathway is an attractive target for anticancer therapies due to its central role in the regulation of proliferation, invasiveness, and survival of tumors [43]. AKT provides a survival signal protecting cells from apoptosis induced by various stresses by multiple mechanisms, such as the phosphorylation of Bad, glycogen synthase-3, and caspase-9 [44]. The PI3K/AKT pathway is frequently altered in human cancers, and an activation of AKT is correlated e.g with invasive and metastasizing breast tumors [45]. In leptin-treated human chondrosarcoma, hepatocellular and endometrial carcinoma cells AKT phosphorylation was found to be increased, and inhibition of PI3K with specific inhibitors abolished leptin-induced proliferation, migration and invasion [35,37,38,42]. Other studies explain the leptin stimulating effect on migration and invasion of various carcinoma cells through the increased expression of various matrix metalloproteinase or integrins via an activation of the nuclear factor κB (NFκB) pathway [34,35]. Moreover, in colon carcinoma cells leptin-induced promotion of motility and invasion was mediated by an activation of PI3K and src kinase pathways resulting in a stimulation of the Rho GTPases rac1, cdc42 and rhoA, proteins known to regulate cell migration by affecting the reorganization of the actin cytoskeleton [29].

### Adiponectin -Effects on immune cells and tumor cells

Adiponectin is a 30 kDa protein secreted exclusively by white adipocytes. Adiponectin circulates as several multimeric species, including a high molecular weight form thought to be the most clinically relevant. Serum levels of adiponectin are markedly decreased in individuals with visceral obesity and states of insulin resistance, such as type 2 diabetes mellitus and artherosclerosis. In contrast to leptin, adiponectin seems to have several beneficial and protective effects. Although its role has not been definitely established, adiponectin was shown to have anti-inflammatory, vasculoprotective, and anti-diabetic effects. Adiponectin is highly abundant in the circulation, with plasma concentrations in healthy humans around 3-30 μg/ml [46], and has a broad spectrum of biological activities. Adiponectin increases the body's sensitivity to insulin including stimulation of glucose uptake in skeletal muscle and suppression of glucose production in liver, and affects the lipid metabolism by decreasing tissue fatty acid content and serum lipids. Interestingly, levels of adiponectin in obese individuals have shown to be decreased even though it comes primarily from adipose tissue. Adiponectin acts through its two receptors, AdipoR1 and AdipoR2, which have unique distributions and affinities for the different forms of circulating adiponectin. AdipoR1 is expressed widely in various tissues, including breast tissue, with the highest level of expression in skeletal muscle, while AdipoR2 is most abundantly expressed in the liver [46].

**Figure 1:**
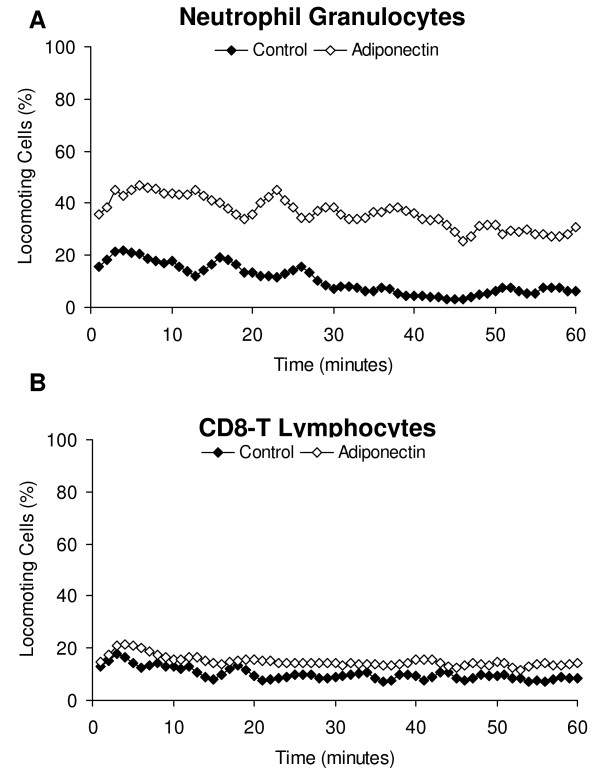
**Effect of adiponectin on the migration of human immune cells**. The adipocytokine adiponectin induced the migratory activity of human neutrophil granulocytes, but did not have any effect on the locomotion of CD8 T lymphocytes. We mixed 2.5 × 10^5 ^neutrophils or T cells with 150 μl of a buffered collagen solution (pH 7.4; 1.67 mg/ml collagen type I) in the presence of 1 μg/ml adiponectin. The buffered collagen suspension was filled into self-constructed chambers and allowed to polymerize for 20 min at 37°C in a 5% CO_2 _humidified atmosphere [9]. We recorded the locomotor activity of the cells by time-lapse videomicroscopy and subsequently analyzed the migratory behaviour by computer-assisted cell tracking. The figure shows mean values of three independent experiments (90 cells were analyzed).

With regard to the immune system, Pang et al. [47] investigated that AdipoR1 is present approximately on 1% of T cells, 93% of monocytes, 47% of B cells, and 21% of NK cells, and the distribution of AdipoR2 was found to be similar. Acting on adaptive immunity, adiponectin inhibits T cell activation and proliferation, although data regarding adiponectin effects on adaptive immune response are relatively spare. In adiponectin-deficient mice, absence of adiponectin was associated with a 2-fold increase in leukocyte-rolling and a 5-fold increase in leukocyte adhesion in the microcirculation [48]. Here, we show for the first time that adiponectin induced the migratory activity of human neutrophil granulocytes within a three-dimensional collagen matrix (from 13% locomoting cells in the control to 39% locomoting cells after stimulation with 1 μg/ml adiponectin; Fig. 1A), whereas it did not have any effect on the migratory activity of human CD8 T lymphocytes. This contradictory results of adiponectin on neutrophil granulocytes could be a consequence of the experimental setup. Whereas in the adiponectin-deficient mice the behaviour of leukocytes during the extravasation process was examined, we investigated in our study the migration of neutrophils, a process subsequently following extravasation. This is of major importance for the interpretation of the results, because participating molecules and molecular mechanisms are different in this course of action. Whereas the extravasation of neutrophils is primarily regulated by the interaction of leukocytes with the endothelium via various adhesion molecules such as selectins or N-cadherin [49], the migratory process is mediated e.g. by the interaction of the cells with the surrounding extracellular matrix via integrins or stimuli-induced rearrangement of the cytoskeleton [50]. In addition, adiponectin inhibits NF-κB activation in endothelial cells and interferes with the function of macrophages; treatment of cultured macrophages with adiponectin markedly inhibit their phagocytotic activity and their production of TNFα in response to lipopolysaccharide stimulation [51]. It suppresses IL-2 enhanced cytotoxic activity of natural killer (NK) cells without affecting basal NK cell cytotoxicity [52]. The exact roles of adiponectin in inflammation and immunity, and especially the underlying molecular mechanism, remain to be defined.

Low serum levels of adiponectin are associated with several metabolic diseases, including obesity and insulin resistance in type-2 diabetes [53]. Moreover, evidence from several studies indicates that serum levels of adiponectin *in vivo *are inversely associated with the risk for multiple cancers, including colon in men [54], prostate [55] as well as breast and endometrium [56,57]. For example, women with a high body mass index and low plasma adiponectin have a risk of endometrial cancer that is 6.5-fold that in women with normal body mass index and higher adiponectin concentrations [58]. Interestingly, the inverse association of adiponectin with breast cancer risk was found solely among postmenopausal women, and not among premenopausal women [56]. In postmenopausal women obesity has been shown to increase rates of breast cancer consistently by 30-50% [59]. Recently, elevated serum adiponectin levels were demonstrated to be independently associated with childhood non-hodgkin's lymphoma (NHL) and poor prognosis. Moreover NHL specimens expressed the adiponectin receptors, suggesting that adiponectin may represent not only a potential clinically significant diagnostic and prognostic marker but also a molecule that may be implicated in NHL pathogenesis [60]. Adiponectin receptors are primarily found to be expressed solely in the malignant and not in the normal control tissue, and the expression can differ by disease stage. For example, in cancerous lung tissue expression of adiponectin receptors was apparent only in the cancerous lung tissue (64.2% AdipoR1 and 61.9% AdipoR2 in cancerous vs. 0% among noncancerous tissue). Specifically, AdipoR1 was expressed in all disease types, but no difference was noted with disease stage, whereas AdipoR2 was mainly expressed in the non-small cell carcinomas and more prominently in the advanced disease stage (80%) [61]. Thus obese individuals with low levels of adiponectin could be at higher risk of developing tumors.

Adiponectin may influence cancer risk through its well recognized effects on insulin resistance, but it is also plausible that it acts on tumor cells directly, because several tumor cell lines express AdipoR1 and AdipoR2. Hence, adiponectin receptors are found to be expressed on breast ([62,63], Table 1), colon [64], and prostate [55] carcinoma cells. Furthermore, adiponectin has been shown to suppress tumor growth in mice, most likely due to inhibition of neovascularization through suppression of endothelial cell proliferation, migration and survival [65]. Several recent studies found that adiponectin decreased cell growth in various breast cancer cell lines such as MCF-7, MDA-MB-231, SK-BR-3 [62,63], inhibited proliferation in prostate cancer cell lines [66], and SW480 colon carcinoma cells (Table 1). In contrast with findings in breast and prostate cancer cell lines, adiponectin stimulated colonic epithelial cell proliferation [67]. This is in accordance with the results in our study, where we observed an enhanced proliferation of various breast carcinoma cell lines, including MDA-MB-468 and MDA-MB-231 upon treatment with adiponectin (Table 1). Also, study of the effects of adiponectin on the apoptosis of cancer cells *in vitro*, which may well differ from the *in vivo *condition, remains inconclusive. In some studies [68,69] apoptosis was increased upon incubation with adiponectin, but this was not observed in other studies [62,66]. These divergent results might be caused by variations in experimental conditions, because especially *in vitro *proliferation studies have limitations; one is the fact that all these studies were performed under serum-free conditions, which may favor an inhibition of cell growth, whereas e.g. in our proliferation assay cells were grown with serum.

To date only two studies have investigated the physiological relevance of the above data by examining the effects of *in vivo *adiponectin administration on tumor growth [65,68]. Animal studies showed that adiponectin supplement therapy could inhibit the development of breast tumors in nude mice, and mice treated with adiponectin displayed a much lower degree of breast tumor metastasis [68]. Although it was not possible to discriminate whether the decreased metastasis was caused by the direct suppressive effects of adiponectin on cell migration and invasion or is simply secondary to the reduced sizes of primary tumors [68], these data clearly support a role of adiponectin in breast cancer progression.

With regard to cell migration we observed differences for the impact of adiponectin on the migratory behaviour of tumor cells. Herein, we show for the first time that adiponectin significantly stimulated the migration of human SW480 colon carcinoma cells, whereas it did not have any effect on the locomotion of various breast carcinoma cells (Table 1). In accordance with the pro-migratory effect on colon carcinoma cells demonstrated herein, very recently two studies report that adiponectin increased the motility of human prostate cancer cells [70] and chondrosarcoma cells [71], too. Although adiponectin research with regard to metastasis is at the beginning, current results on its effects on the migratory and proliferative activity of carcinoma cells indicate a distinct role of this adipocytokine in cancer pathogenesis.

### Adiponectin

#### Signaling pathways linking adiponectin with carcinogenesis

Despite the mounting experimental data, the molecular mechanisms through which adiponectin mediates its effects have not been fully elucidated. Possible mechanisms, especially for its antiproliferative effects, have been reviewed in detail by Kelesidis et al. [46]. Adiponectin, via its specific receptors, mediates several signaling pathways such as 5'-AMP-activated protein kinase (AMPK), peroxisome-proliferators-activated receptor (PPAR)-α and p38 mitogen-activated protein (MAP) kinase. Several studies strongly suggest that both the antiproliferative and pro-apoptotic effects of adiponectin are mediated by AMPK [69,72]. Activation of AMPK inhibits enzymes that regulate protein, fatty acid, and triglyceride synthesis, including mammalian target of rapamycin (mTOR), fatty acid synthase, and glycerol phosphate acyltransferase [73]. In addition, activated AMPK positively regulates two important proteins for the control of growth arrest and apoptosis, p53 and p21 [73]. In MCF-7 breast carcinoma cells, adiponectin-mediated antiproliferative responses were also accompanied by an inhibition of MAP kinase pathway [69], which is known to be associated with a decrease of cell proliferation e.g. in human osteoblasts. More recently, c-Jun NH2-terminal kinase (JNK) and STAT-3 were also shown to be downstream effectors of adiponectin [74]. It has been demonstrated that adiponectin stimulates JNK activation, which is involved in the regulation of cell proliferation and apoptosis during various physiological and pathological events, such as tumor development [75]. STAT-3 also regulates cellular functions such as cell proliferation, survival, differentiation as well as apoptosis, and dysregulation of the STAT system directly contributes to malignant transformation and cancer progression [76]. Adiponectin was shown to stimulate JNK activation in prostate cancer DU145, PC-3, and LNCaP-FGC cells, hepatocellular carcinoma HepG2 cells, and C2C12 myoblasts, but also drastically suppress constitutive STAT-3 activation in DU145 and HepG2 cells [74]. This suggests that JNK and STAT-3 may constitute a universal signaling pathway to mediate adiponectin's pathophysiological effects on metabolic syndrome and the pathogenesis of cancer. Very recently, adiponectin was identified to reduce mRNA levels of genes involved in cell cycle regulation (MAPK-3, ATM) and apoptosis (BAG-1, BAG-3, MX-1, TP53) [77].

With regard to the molecular mechanisms mediating the pro-migratory effects of adiponectin the investigations have just started. Very recently, adiponectin was shown to increase the motility of human prostate cancer [70] and chondrosarcoma cells [71] via a transcriptional upregulation of integrins and activations of p38, AMPK, and NFκB-cascades. In endothelial progenitor cells adiponectin promoted the migration activities mainly through the phosphorylation of AKT and the activations of Cdc42 and Rac1 [78]. In summary, although an increasing number of studies confirm and improve the existing findings, there is more to be studied to better understand the mechanisms underlying the antiproliferative as well as the pro-migratory effects of adiponectin.

## Conclusions

The cause of cancer has seemingly been pinned down to be due to fundamental aberrations of cellular functions, which also include many aspects of molecular biology. The pathway of tumor formation is a multistep journey including at least six essential alterations in cell physiology that collectively dictate malignant growth and the spread of transformed cell clones [79]. Besides these alterations which have a genetic basis, tumor tissue is not to be regarded as an independent entity isolated and set apart from the body's environment, but is highly dependent upon a multitude of factors that have been suggested to promote and impair cancer progression. A prerequisite for tumor cell invasion and metastasis formation is the capability of malignant cells for active migration. This cellular process is regulated by a myriad of external signals such as chemokines and neurotransmitters [8].

Obesity is related to a condition of chronic inflammation characterised by abnormal production of inflammatory cytokines with local effects e.g. TNFα or systemic effects e.g. IL-6, that can contribute to the pathogenesis of malignant diseases. Chronic inflammation induced by obesity can affect both tumor initiation and tumor progression, such as adipocyte-conditioned medium can e.g. promote tumor migration [36]. Balkwill and Mantovani's study elaborated on Virchow's theory of a connection between inflammation and cancer [80], in which they describe the attraction of leukocytes toward tumor cells, initiated through tumor-released chemokines; they likewise postulated the attraction of tumor cells by other chemokine-producing cells. In recent years, improved understanding of the inflammatory microenvironment of malignant tissues has supported this hypothesis. Furthermore, previous studies of our group have extended a functional interplay between cancer progression and its environment, demonstrating a strong impact of the nervous system on the migration of leukocytes and tumor cells, too [8]. Apart from the effect they inherently have on leukocytes causing activation as well as migration of these cells, they also mediate the neo-angiogenesis of malignant tissue, and regulate directed metastatic migration. The effects of adipocytokines on the migration of tumor cells, which are herein reviewed, give further credence to Balkwill and Mantovani's hypothesis. Accordingly, as summarized in Table 2, leptin and adiponectin are shown to participate in the process of carcinogenesis, including the migration of tumor cells, and in the immune response. Leptin and adiponectin, the most abundant adipocytokines produced by adipocytes and the best studied members of this family, stimulate growth, migration and invasion of cancer cells *in vitro*, thus displaying a capacity for promoting malignant biological behavior of cancer. Interestingly, leptin and adiponectin show *in vivo *strongly varying serum concentrations. Leptin concentrations *in vivo *are high in obese patients, whereas adiponectin levels in the blood decrease with body mass index and leptin increases. Consequently, investigating the effectiveness of these adipocytokines alone delivers different results on tumor cells than in presence of more than one adipocytokine. For example, adiponectin was shown to inhibit leptin-induced cell proliferation in preneoplastic colon epithelial cells by inhibiting leptin-induced NFκB-dependent autocrine IL-6 production and trans-IL-6 signaling. Thus adiponectin may be an important regulator of colon epithelial cell homeostasis by linking the observed reduced risk for cancer in populations with high serum adiponectin concentrations to specific mechanism of cell number homeostasis [81]. Recently, we could demonstrate in our own studies, that adiponectin and leptin alone significantly stimulated the migration of SW480 colon carcinoma cells, respectively, whereas the combination of both negated these pro-migratory effects (data not shown). Taken together, these results suggest that the balance in the concentrations of adipocytokines such as leptin and adiponectin ultimately determines the consequences of these substances for the immune response and the tumor progression. However, the exact molecular mechanisms of these adipocytokines are still unsolved, and it is unlikely that there is a "one system fits all" mechanism for. Thus, further studies illuminating the signaling pathways of the single adipocytokines, but also possible crossregulations with intracellular cascades activated by other more recently discovered adipocytokines, hormones, cytokines or growth factors, will be needed.

**Table 2:** Summary of the general and specific function of adipocytokines

Molecule	Function/effect in general	Leukocyte function	References	Function and cancer type	References
**Leptin**	Satiety and appetite; signals to the brain to regulate energy homoeostasis and body weight.	Chemoattractant of neutrophils, monocytes & macrophages; stimulates chemokinesis of eosinophils	[18,20-22]	Promotes migration & invasion of chondrosarcoma, glioma, colon, endometrial prostate & hepatocellular carcinoma cells	[28,29,34-38]
**Adiponectin**	Regulator of energy homoeostasis: enhances insulin sensitivity and glucose uptake; has anti-inflammatory properties.	Stimulates the migration of neutrophils & inhibits phagocytotic activity of macrophages.	[51] Figure 1	Suppresses growth in breast, colon & prostate cancer cell lines Stimulates the migration of chondrosarcoma, colon & prostate carcinoma cells	[62,63,67,70,71] Table 1

In summary, this review clarifies the enormous wide range of efficacy adipocytokines such as leptin and adiponectin do have *in vivo *on leukocytes and tumor cells besides their already known physiological functions. The pathophysiological and biological mechanisms underpinning these associations are only starting to be understood. There is a necessity to further investigate and characterize their modes of action that link obesity and inflammation as well as cancer for future design of novel therapeutics for the treatment but also prevention of obesity-associated carcinoma without hindering the immune response.

## Abbreviations

ADSF: adipocyte-secreted factor; AMPK: 5'-AMP-activated protein kinase; bFGF: basic fibroblast growth factor; CCL2: CC-chemokine ligand 2; EGFR: epidermal growth factor receptor; ERK: extracellular signal-regulated kinase; FIZZ3: found in inflammatory zone 3; G-CSF: granulocyte colony-stimulating factor; IFN-gamma: interferon gamma; IL: interleukin; IGF-I: insulin-like growth factor-I; JAK: Janus kinase; JNK: c-Jun NH2-terminal kinase; LIF: leukaemia inhibitory factor; LPS: lipopolysaccharides; MAP: mitogen-activated protein; mTOR: mammalian target of rapamycin; PBMNLs: polymorphonuclear leucocytes; PI3K: phosphatidylinositol-3-kinase; PPARα: peroxisome-proliferators-activated receptor α; TGF-beta1: transforming growth factor-beta1; TNF-α: tumor necrosis factor alpha; VEGF: vascular endothelial growth factor.

## Competing interests

The authors declare that they have no competing interests.

## Authors' contributions

JR performed the experiments and surveyed literature of this review, and KL selected the literature and wrote the manuscript. The authors read and approved the final manuscript.

Search strategy and selection criteria

We selected data for this review by searches of PubMed, using the search terms: "cancer"/"tumo(u)r" and "migration"/"invasion"/"metastasis" as well as "proliferation", or "leukocytes"/"lymphocytes"/"immune cells"/"neutrophils" and "migration"/"motility", combined with "adipocytokines" or "leptin" and "adiponectin", respectively. Only papers published in English within the past 12 years were included.
